# Development of OXY111A, a novel hypoxia-modifier as a potential antitumor agent in patients with hepato-pancreato-biliary neoplasms - Protocol of a first Ib/IIa clinical trial

**DOI:** 10.1186/s12885-016-2855-3

**Published:** 2016-10-19

**Authors:** Perparim Limani, Michael Linecker, Philipp Kron, Panagiotis Samaras, Bernhard Pestalozzi, Roger Stupp, Alexander Jetter, Philipp Dutkowski, Beat Müllhaupt, Andrea Schlegel, Claude Nicolau, Jean-Marie Lehn, Henrik Petrowsky, Bostjan Humar, Rolf Graf, Pierre-Alain Clavien

**Affiliations:** 1 Swiss Hepato-Pancreato-Biliary (HPB) and Transplantation Center, University Hospital Zurich, Raemistrasse 100, Zurich, CH-8091 Switzerland; 2 Department of Surgery, University Hospital Zurich, Raemistrasse 100, CH-8091 Zurich, Switzerland; 3 Department of Oncology, University Hospital Zurich, Raemistrasse 100, Zurich, CH-8091 Switzerland; 4 Department of Clinical Pharmacology and Toxicology, University Hospital Zurich, Raemistrasse 100, Zurich, CH-8091 Switzerland; 5 Department of Gastroenterology and Hepatology, University Hospital Zurich, Raemistrasse 100, Zurich, CH-8091 Switzerland; 6 Friedman School of Nutrition Science and Policy, 150 Harrison Ave, Boston, MA 02111 USA; 7 Institut de Science et d’Ingénierie Supramoléculaires (ISIS), Université de Strasbourg, 8 allée Gaspard Monge, Strasbourg, F-67000 France

**Keywords:** Hypoxia, Hepatopancreatobiliary tumor, Colorectal cancer, Hepatocellular carcinoma, Cholangio carcinoma, Myo-inositol trispyrophosphate, ITPP

## Abstract

**Background:**

Solid tumors, such as hepato-pancreato-biliary cancer, develop tumor hypoxia with tumor growth. Despite advances in surgery, a majority of these patients are in an unresectable condition. At this stage standard cytotoxic chemotherapy regimens are applied with limited success. Novel biological treatment options based on an antiangiogenic mechanism of action neglect other hypoxia mediated mechanisms (e.g. epithelial-mesenchymal transition, Warburg effect, and immunological response) leading to an increased invasiveness with a poor outcome.

The novel antihypoxic molecule myo-inositoltrispyrophosphate (ITPP, OXY111A) acts as an allosteric effector of hemoglobin and promotes normoxia in hypoxic tumors. In preclinical studies, tumor growth was reduced and survival prolonged. Additionally, a beneficial side effect profile was observed.

**Methods:**

In this first Ib/IIa clinical trial we will assess safety and tolerability of OXY111A as well as a proof of concept regarding efficacy in patients with non-resectable primary and secondary tumors of the liver, pancreas, and biliary tract. The study design is exploratory, prospective, open-labelled and mono-centric. The study is divided in a dose escalation part with a maximum of 48 subjects and an extension part, in which 21 subjects will be included.

**Discussion:**

The novel antihypoxic compound OXY111A has been tested in several cancer animal models showing beneficial effects for both survival and low side effect profiles. This first in patient application of OXY111A will reveal potential beneficial outcomes if anti-hypoxic therapy is added to standard cytotoxic treatment in patients with primary and secondary hepatopancreatobiliary tumors.

**Trial registration:**

Institution Ethical Board Approval ID: KEK-ZH-Nr. 2014-0374; Swiss regulatory authority Swissmedic (2015DR1009); ClinicalTrials.gov Identifier: NCT02528526, prospectively registered on November 11^th^, 2014.

## Background

Hypoxia occurs in almost any solid tumor beyond a certain size and it crucial for the development and progression of the disease [1]. The decreased oxygen partial pressure modifies tumor behavior. Best known is the increase in cancer-associated angiogenesis following hypoxia. Novel anti-cancer agents (e.g. bevacizumab) have been designed to specifically target the angiogenic response with the aim to starve the tumor. However, angiogenesis is only one part of the tumor's response towards hypoxia [2].

**Fig. 1:**
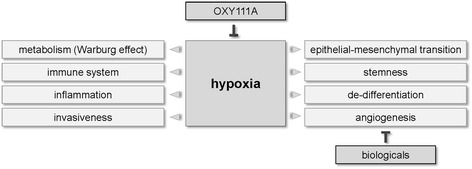
Effects of hypoxia in HPB tumors

Molecularly, hypoxia leads to the stabilization of HIF (Hypoxia-Inducible Factors), transcription factors that regulate the response of cancer cells to tumor hypoxia. Apart from angiogenesis (e.g. via vascular endothelial growth factor, VEGF), HIFs promote a number of processes known to foster tumor development, including inflammation (e.g. via the NF-κB pathway), a shift to glycolysis (Warburg effect, e.g. via up-regulation of glucose transporter), invasive behavior (e.g. via Twist, an inducer of the epithelial-mesenchymal transition), and malignant potential (e.g. via OCT4 and other genes promoting a stem cell phenotype). Furthermore, HIFs have been implicated in the suppression of adaptive immunity and the protection from cell death. Importantly, the presence of hypoxia predicts a bad outcome for many tumor types, consistent with the observation promoting aggressive cancer behaviour (Fig. 1) [3]. Therefore, the prevention of hypoxia in the first place may provide an anticancer strategy superior to existing anti-angiogenic approaches [2, 4].

OXY111A is a synthetic allosteric effector of hemoglobin 4. OXY111A is taken up by red blood cells via the band 3.1 transporter and promotes the dissociation of oxygen from hemoglobin under conditions of low pO2. Thus, OXY111A increases the oxygen-releasing capacity of hemoglobin specifically under hypoxic conditions [5] and has the potential to prevent hypoxia without affecting the oxygen levels in the surrounding normoxic cells. OXY111A is the first anti-hypoxic compound without known, significant toxicities.

Thus far, OXY111A has been tested in five animal models of cancer [6–10]. In a syngeneic, orthotopic rat model of hepatocellular carcinoma, OXY111A prevented HIF1α stabilization as well as VEGF production and increased the apoptotic index, leading to a rapid reduction in tumor load along with dramatically improved survival in treated animals. Consistent with this finding, OXY111A treatment is generally associated with the inhibition of the hypoxic tumor response in all models studied [5–10]. The concept of vessel normalization through OXY111A has been impressively confirmed in a syngeneic rat model of pancreatic carcinoma and is of direct relevance to our patients [9]. Particularly important is the potentiation of chemotherapy, which is very likely a consequence to vessel normalization. Notably, no side effects were detected in all animal models, including those where OXY111A was shown to improve exercise capacity in healthy and sick mice [11].

The aim of this phase Ib/IIa clinical is to evaluate safety and tolerability of OXY111A in order to determine a maximum tolerated dose (MTD) as well as a proof of concept regarding efficacy in patients with primary or secondary malignancies of the liver, pancreas and biliary tract.

## Methods/Design

### Study design

This is a phase Ib/IIa dose escalation and extension study on the effects of OXY111A in patients suffering from primary and secondary hepato-pancreato-biliary or gastrointestinal malignancies not curable by surgical means.

**Fig. 2:**
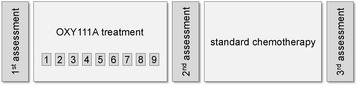
Study flow chart

The study design is exploratory, prospective, open-labelled and mono-centric. The study is divided in a dose escalation part with a maximum of 48 subjects and an extension part, in which 21 subjects will be included (specifically 4 dose expansion groups of 6 patients each determined by histology). Overall duration of an individual subject’s participation in the study is minimum 4 and maximum 9 weeks (Fig. 2).

### Dose escalation and extension

The aim of the escalation study part is the assessment of additional preliminary safety and tolerability data for the MTD. Safety will be assessed according to the (Common Terminology Criteria for Adverse Events (CTCAE), also known as common toxicity criteria (CTC). These criteria are issued by the US National Cancer Institute for the standardized classification of adverse effects of drugs used in cancer therapy. Specific symptoms and conditions define each level of this classification system ranging from 1-5, where 1 is the mildest form of the specific symptom and 5 is death. The CTCAE version used for this study will be version 4.03.

According to CTCAE, Dose Limiting Toxicity (DLT) will be defined as:

- any grade 5 toxicity
- any grade 4 neutropenia lasting >7 days
- any grade 3 febrile neutropenia, thrombocytopenia, or anemia
- any grade 3 or 4 nonhematologic toxicity, excluding alopecia, that is considered by the investigator to be definitely, probably or possibly related to OXY111A.

A significant Treatment-Emergent Toxicity (TET) is defined as any other grade 2 toxicity considered by the investigator to be definitely, probably, or possibly related to OXY111A, but not fulfilling the criteria for DLT.

Based on the assessment of DLT and TET the MTD will be noted. MTD is defined as the dose preceding the dose level at which one patient in the cohort experiences a DLT during the treatment cycle, or the dose level at which 3 or more patients experience a significant TET.

There are two factors to be considered for the OXY111A dose escalation part of this study: total dose administered and infusion rate. The human equivalent dose-corrected No Observed Adverse Effect Level (NOAEL) in rats was 15’000 mg/m^2^ once a week for 5 weeks, while it was 43’750 mg/m^2^ in minipigs also once weekly (unpublished data obtained from Normoxys®). From animal studies, hypernatremia is known to be associated with high plasma concentrations typically encountered at the end of the OXY111A infusion and probably associated with infusion rate. Therefore, infusion rates of not more than 750 mg/m^2^/min are recommended. According to available phase I data in healthy volunteers (unpublished data obtained from Normoxys®), cohort 1 starts at a dose close to 5’600 mg/m^2^ weekly, i.e. 1866 mg/m^2^ thrice weekly. Dose escalation is performed according to the following scheme up to a maximum calculated dose of 43’700 mg/m^2^ weekly, which corresponds to the NOAEL level administered once weekly in minipigs.

Table 1 schedules dose escalation. In each cohort, 3 patients will be included (Table 1).

**Table 1:** Dose escalation scheme

Cohort No.	Weekly Dose	Single Dose	Total Dose (9 applications )	% Dose Escalation
1	5'598 mg/m^2^	1'866 mg/m^2^	16'794 mg/m^2^	baseline
2	11'196 mg/m^2^	3’732 mg/m^2^	33'588 mg/m^2^	100 %
3	16'800 mg/m^2^	5‘600 mg/m^2^	50‘400 mg/m^2^	50 %
4	21‘000 mg/m^2^	7‘000 mg/m^2^	63‘000 mg/m^2^	25 %
5	26‘250 mg/m^2^	8‘750 mg/m^2^	78‘750 mg/m^2^	25 %
6	31‘500 mg/m^2^	10‘500 mg/m^2^	94‘500 mg/m^2^	20 %
7	37‘170 mg/m^2^	12'390 mg/m^2^	111‘510 mg/m^2^	18 %
8	43'500 mg/m^2^	14'500 mg/m^2^	130'500 mg/m^2^	~17 %

No more than 6 patients (3 + 3 patients) will receive the same dose during the dose escalation phase according to the following rules.

1. Dose escalation to the subsequent cohort is possible if there is no DLT and no significant TET.
2. If 1 DLT occurs, the ongoing cohort will be stopped, and another three patients will receive the next lower dose level tested, if only 3 patients have received this lower dose. If already 6 patients have received this lower dose, this lower dose represents MTD. If DLT occurs in cohort 1, the dose will be reduced to 4’200 mg/m^2^ weekly (ie. to 75 %), and this dose will be tested in 3 patients.
3. If a 1-2 significant TETs occur, another three patients will receive the same dose.
4. If out of 6 patients with the same dose, 3 or more significant TETs occur, this dose level will be the MTD.
5. If out of 6 patients, 1-2 experience significant TETs, the dose may be increased to the next dose escalation step as shown in Table 1 in 3 additional patients. However, if a DLT has been observed, this dose must not be administered again.

Following these rules, a maximum of 48 patients are included in the dose escalation phase of the study. As soon as the MTD during the “dose escalation” phase is defined, a “dose extension” is performed in additional 21 subjects, who will receive the MTD.

In parallel, a maximum of three patients receiving the same dose of the IMP will be included in the study. Depending on the safety and tolerability of the compound, the next patient (or maximal the next three patients) who receives a higher dose, will be included after the last patient completed the whole IMP cycle (with a total of 9 applications) receiving the last lower dose.

A classical 3 + 3 phase I design was chosen for the dose escalation part of the study, because there is insufficient information on safety and tolerability of doses above 5’600 mg/m^2^ once weekly and of repeated doses in humans (unpublished data obtained from Normoxys®). This design induced the least toxicities in a simulation study comparing various dose finding escalation strategies and therefore appears to be most appropriate for a study using such substance in terminally ill cancer patients, where the therapeutic benefit is yet unknown.

The infusion rate must not be above 750 mg/m^2^/min. In order to achieve this, a constant infusion rate at 1/10 of the NOAEL infusion rate is chosen: an infusion rate of 75 mg/m^2^/min is scheduled for all dose steps. Due to the short half-life of OXY111A and the proposed mechanism of action, a slow, constant infusion over several hours appears more promising than a short infusion leading to excessively high concentrations of the compound.

### Study population and number of patients

Patients with primary and secondary hepato-pancreato-biliary and gastrointestinal neoplasia will be assessed for eligibility at the interdisciplinary tumor board of the Swiss HPB Center. Patient information and informed consent will be obtained in the outpatient and inpatient hospital setting. Overall, a maximum of 69 patients will be included consisting of maximum of 48 in the dose escalation and 21 patients in the extension part of the study. OXY111A will be administered in an outpatient setting in the Phase I unit of the Clinical Trials Center of the University Hospital Zurich, in which the study equipment is reserved for this study explicitly. Appropriate back up material will be available in case of technical dysfunction. The study is planned to be completed within approximately two years.

### Inclusion criteria

Participants fulfilling all of the following inclusion criteria are eligible for the study:

- Informed Consent as documented by signature
- Patients diagnosed for non-resectable hepato-pancreato-biliary or gastrointestinal neoplasm
- Male and Female patients ≥ 18 years of age
- Eastern Cooperative Oncology Group (ECOG) performance status score of ≤ 2 at study entry.
- A life-expectancy of >3 months
- Adequate hematologic function, as defined by
  - ○ an absolute neutrophil count of >1000/mm^3^
  - ○ a hemoglobin level of >10 g/dL
  - ○ a platelet count of >100,000/mm^3^
- Adequate renal function, as defined by a serum creatinine level of ≤1.5 x the upper normal limit.
- Use of effective contraception. Female patients who are surgically sterilized/hysterectomized or post-menopausal for longer than 2 years are not considered as being of procreative potential.
- Adequate recovery from recent surgery, chemotherapy and radiation therapy. At least 28 days must have been elapsed from major surgery, prior chemotherapy, prior treatment with an investigational agent or device, or prior radiation therapy (palliative radiation therapy is allowed).
- Accessible for treatment and follow-up. Patients enrolled in this trial must be treated at the participating center.

### Exclusion criteria

The presence of any of the following exclusion criteria will lead to exclusion of the potentially eligible participant:

- Contraindications to the class of drugs used in this study, e.g. known hypersensitivity or allergy to class of drugs or the investigational product
- Women who are pregnant or breast feeding
- Intention to become pregnant during the course of the study
- Lack of safe contraception, defined as: Female participants of childbearing potential, not using and not willing to continue using a medically reliable method of contraception for the entire study duration, such as oral, injectable, or implantable contraceptives, or intrauterine contraceptive devices, or who are not using any other method considered sufficiently reliable by the investigator in individual cases. Please note that female participants who are surgically sterilised / hysterectomised or post-menopausal for longer than 2 years are not considered as being of child bearing potential.
- Other clinically significant concomitant disease states (e.g., renal failure, hepatic dysfunction, cardiovascular disease, etc.)
- Known or suspected non-compliance, drug or alcohol abuse
- Inability to follow the procedures of the study, e.g. due to language problems, psychological disorders, dementia, etc.
- Participation in another study with investigational drug within the 30 days preceding and during the present study
- Previous enrolment into the current study
- Enrolment of the investigator, his/her family members, employees and other dependent persons.

### Assessment of primary outcome

The primary outcome, safety and tolerability (establishing MTD) of the investigational medical product (IMP) will be assessed at every application for each patient. According to the 3 + 3 dose escalation schedule, after each patient, a decision on the continuation will be taken, i.e. to either add an additional patient in the same cohort or to continue with the next cohort after three patients.

Assessment will be done using CTCAE. The obtained data will be added to the electronic case report form (eCRF) using the software secuTrial® (interActive Systems GmbH (iAS), Berlin/Germany).

### Assessment of secondary outcomes

Secondary outcome focuses on efficacy as measured by means listed below:

- Imaging assessment by 18-Fluorodeoxyglucose and Positron Emission Tomography (18 F-FDG PET) in FDG-avid tumors and Magnetic resonance imaging (MRI) in non-FDG avid tumors.
  - ○ 18F-FDG PET: Response evaluated by Response Evaluation Criteria In Solid Tumors (RECIST) 1.1 and European Organization for Research and Treatment of Cancer (EORTC) criteria
  - ○ MRI: Response evaluated by RECIST 1.1.
- Peripheral blood serum for safety and efficacy (e.g. tumor markers)
- Tumor biopsy if possible for histopathological examination.

### Pharmacokinetics

Blood samples are taken before OXY111application as well as at 3 and 6 hours after application start and 30 minutes, 1, 2 and 40 hours following application. Plasma levels of OXY111A are measured by Swiss BioAnalytics AG (Birsfelden, Switzerland) under ICH Good Laboratory Practice (GLP) conditions and validated by Food and Drug Administration (FDA) and European Medicines Agency (EMA) guidance [12].

### Ethics

This study is conducted under the principles of the Declaration of Helsinki and International Conference on Harmonisation E6 Good Clinical Practice (ICH E6 GCP) guidelines. The study protocol has been approved by the independent medical ethics committee of canton Zurich (Kantonale Ethikkommission Zürich, Switzerland, KEK-ZH-Nr. 2014-0374) and the Swiss regulatory authority Swissmedic (2015DR1009). The study protocol has been registered on an international primary trial register (ClinicalTrials.gov Identifier: NCT02528526).

## Discussion

The aim of this trial is to assess the novel antihypoxic compound OXY111A in patients with non-resectable primary and secondary tumors of the liver, pancreas and biliary tract. Hepato-pancreato-biliary and gastrointestinal tumors, as known for solid tumors, develop hypoxia with consecutive modification of hypoxia dependent pathways [1]. Despite several advantages in drug development regarding tumor angiogenesis, many other hypoxic mechanisms have not been addressed so far [2]. The novel antihypoxic compound OXY111A has been tested in several cancer animal models showing beneficial effects for both survival and low side effect profiles [6–10].In this phase Ib/IIa clinical trial, application of OXY111A will be evaluated in patients with primary and secondary HPB tumors focussing on safety and tolerability, as well as efficacy.

## Abbreviations

18F-FDG PET: 18-Fluorodeoxyglucose and Positron Emission Tomography
CT: Computed Tomography
CTC: Common Toxicity Criteria
CTCAE: Common Terminology Criteria for Adverse Events
DLT: Dose Limiting Toxicity
ECOG: Eastern Cooperative Oncology Group
eCRF: electronic Case Report Form
EMA: European Medicines Agency
EORTC: European Organization for Research and Treatment of Cancer
FDA: Food and Drug Administration
HIF: Hypoxia-Inducible Factors
ICH E6 GCP: International Conference on Harmonisation E6 Good Clinical Practice
ICH GLP: International Conference on Harmonisation Good Laboratory Practice
IIT: Investigator Initiated Trial
IMP: Investigational Medical Product
ITPP: Myo-inositol trispyrophosphate
MRI: Magnetic resonance imaging
MTD: Maximum Tolerated Dose
NF-κB: nuclear factor 'kappa-light-chain-enhancer' of activated B-cells
NOAEL: No Observed Adverse Effect Level
OCT4: Octamer binding transcription factor 4
OXY111A: Study drug name of the generic myo-inositol trispyrophosphate
pO_2_: Oxygen partial pressure
RECIST: Response Evaluation Criteria In Solid Tumors
TET: Treatment- Emergent Toxicity
VEGF: Vascular Endothelial Growth Factor
